# Regulation of soybean drought response by mepiquat chloride pretreatment

**DOI:** 10.3389/fpls.2023.1149114

**Published:** 2023-05-08

**Authors:** Xiyue Wang, Xinyu Zhou, Zhipeng Qu, Chao Yan, Chunmei Ma, Jun Liu, Shoukun Dong

**Affiliations:** ^1^ College of Agriculture, Northeast Agricultural University, Harbin, China; ^2^ Lab of Functional Genomics and Bioinformatics, Institute of Crop Science, Chinese Academy of Agricultural Sciences, Beijing, China

**Keywords:** drought stress, mepiquat chloride, soybean, molecular mechanism, physiology, growth characteristic

## Abstract

**Introduction:**

Soybean is the world’s most important cultivated crop, and drought can affect their growth and, eventually, yields. Foliar application of mepiquat chloride (MC) can potentially alleviate the damage caused by drought stress in plants; however, the mechanism of MC regulation of soybean drought response has not been studied.

**Methods:**

This study investigated the mechanism of soybean drought response regulation by mepiquat chloride in two varieties of soybean, sensitive Heinong 65 (HN65) and drought-tolerant Heinong44 (HN44), under three treatment scenarios, normal, drought stress, and drought stress + MC conditions.

**Results and discussion:**

MC promoted dry matter accumulation under drought stress, reduced plant height, decreased antioxidant enzyme activity, and significantly decreased malondialdehyde content. The light capture processes, photosystems I and II, were inhibited; however, accumulation and upregulation of several amino acids and flavonoids by MC was observed. Multi-omics joint analysis indicated 2-oxocarboxylic acid metabolism and isoflavone biosynthetic pathways to be the core pathways by which MC regulated soybean drought response. Candidate genes such as *LOC100816177, SOMT-2, LOC100784120, LOC100797504, LOC100794610*, and *LOC100819853* were identified to be crucial for the drought resistance of soybeans. Finally, a model was constructed to systematically describe the regulatory mechanism of MC application in soybean under drought stress. This study fills the research gap of MC in the field of soybean resistance.

## 1 Introduction

Soybean (*Glycine max*) is one of the most significant crops currently grown, which also serves as the primary raw material for vegetable oil and protein production (Liu et al., 2020). Soybeans contain a range of phytochemicals, including phenolic compounds and isoflavones; additionally, they are considered to be a traditional health food owing to their high nutritional value (Wang and Komatsu, 2017). Moreover, they are crucial in animal feed production (Tyczewska et al., 2016). Global soybean productivity and yield per hectare have consistently increased over the past century because of advances in agronomic practices and cultivars, which are suited for different latitudes (Ainsworth et al., 2012). However, there is further scope for improving the breeding of soybeans to increase their yield. To date, hybridization, and molecular breeding have been conducted as part of soybean breeding (Cao et al., 2022). However, certain environmental stressors, such as drought, salinity, and extremely high temperatures, have been found to severely restrict plant spread, affect growth and development, and lower crop output (Zhang et al., 2022).

Drought is one of the major abiotic stresses that prevents plant growth and productivity and jeopardizes the sustainability of crop production. It hinders plant growth by interrupting water flow and reducing water availability (Dubey et al., 2021). Notably, at the cellular and organismal levels, plants have developed physiological and biochemical responses to drought (Razi and Muneer, 2021). Plants under drought stress have yellowed and curled or wilted leaves. Additionally, drought stress affects the morphology of the roots, resulting in a decrease in root length, area, and diameter (Bashir et al., 2019). According to the physiological theory, drought boosts antioxidant capacity, antioxidant enzyme activity, and osmotic adjustment substance accumulation in plants (Wang et al., 2022a). Furthermore, plant hormones significantly influence the response of plants to drought stress. For example, drought stress results in frequent accumulation of abscisic acid (ABA) (Waadt et al., 2022). Additionally, dryness can harm the plants’ ability to synthesize oxygen, typically showing a reduction in photosynthetic capacity and chlorophyll fluorescence characteristics (Yang et al., 2019). Studies on drought have advanced beyond the physiological level and presently include a thorough examination of molecular mechanisms. In our earlier study, we discovered a drought-resistant system focusing on the glycolysis pathway, tricarboxylic acid (TCA) cycle, and pentose phosphate pathway, as well as potential candidate genes for drought resistance (Wang et al., 2022b). Understanding the mechanism of drought can enable researchers to improve the drought resistance of plants using certain techniques, including gene editing and hybridization technology. However, breeding of high-resistance varieties usually requires long periods of time and is expensive.

Currently, several exogenous chemicals, such as melatonin, are typically used to enhance plant growth and stress resistance (Tiwari et al., 2021). Mepiquat chloride (MC), an endogenous plant growth inhibitor, is commonly used in cotton cultivation. Slowing down the activity of gibberellins (GAs) involved in cell elongation prevents activation of signaling pathways, inhibits vegetative growth, and disrupts GA homeostasis by activating site-specific genes, finally resulting in dwarf plants (Tung et al., 2020). Additionally, our previous study demonstrated that MC prevents soybean development by preventing the production of GA, zeatin, brassinolide, and other plant hormones, as well as genes relevant to cell wall construction and signaling pathways; further, we discovered that MC may be resistant to drought. MC use in typical water circumstances resulted in a significant amount of flavonoid accumulation and increased amount of ABA at the hormonal level (Wang et al., 2022c). Additionally, several studies revealed that MC improves stress resistance. For example, Liu et al. (2018) found that the use of MC can improve the physiological metabolic function of plants by increasing the chlorophyll and soluble protein content in sunflower leaves, increasing the accumulation of free amino acids, decreasing malondialdehyde (MDA) production, and enhancing the activities of catalase (CAT), peroxidase (POD), and superoxide dismutase (SOD) protective enzymes. Luo et al. (2010) demonstrated that MC treatment significantly regulated the cold tolerance and growth of sweet pepper, as evidenced by an increase in SOD and CAT activities, increase in osmotic adjustment compounds such as proline and soluble sugar in leaves, and a further accumulation of ABA, which reduced the damage caused by low-temperature stress. However, the potential molecular mechanism of mepiquat chloride regulation of soybean drought response remains unknown.

In this study, the effects of MC on the growth and physiology of soybean under drought stress were systematically reported by applying MC to leaves and simulating drought with Polyethylene Glycol (PEG-6000). The molecular mechanism of MC regulating soybean response to drought was further analyzed, which filled the research gap of MC in the field of soybean stress resistance and laid a foundation for the promotion of MC in soybean production.

## 2 Materials and methods

### 2.1 Experimental materials and experimental design

Two varieties, sensitive Heinong 65 (HN65) and drought-tolerant Heinong 44 (HN44), were selected as the experimental materials because of their distinct drought tolerances at the seedling stage (Wang et al., 2022d). The sand culture method was used for the experiment. Eight seeds were sown per pot and thinned to three seedlings per pot upon full development of true leaves. They were watered once daily with distilled water (500 mL) until the true leaves were fully developed, and subsequently with Hoagland nutrient solution (500 mL) once daily until the true leaves were fully expanded. The following treatments were introduced when the soybeans reached the seedling stage (V3): Treatment 1 (normal conditions), normal water conditions were maintained by adding 500 mL of Hoagland nutrition solution daily. Treatment 2 (drought), normal water conditions were maintained for the first 3 days, and the drought stress treatment was applied subsequently. A Hoagland nutritional solution (500 mL) containing 15% PEG-6000 was applied once daily for the following 4 days. Treatment 3 (drought + MC): MC solution (100 mg/L) was sprayed evenly on all leaves until the leaves were completely wet but not dripping. The normal water conditions were maintained for the first 3 days. Subsequently, a drought stress treatment was applied, and for the following 4 days, 500 mL of a nutritional solution containing 15% PEG-6000 was applied once a day. All treatments were then sampled and measured. To ensure sufficient experimental materials, each treatment retained 30 pots, maintaining more than three biological replicates (including morphological and physiological measurements). To comprehend the regulation of drought by MC at the molecular level, transcriptome and metabolome analysis were performed in treatments 2 and 3, including two groups, HN44 Drought vs. HN44 Drought + MC and HN65 Drought vs. HN65 Drought + MC (Hereafter HN44 Group and HN65 Group, respectively).

### 2.2 Determining morphological and physiological indices

Plant height was measured by a meter scale. For leaf and stem dry weight, the plant was cut at the cotyledon scar, and the leaves and stems were manually separated and placed in a paper envelope, which was then sealed. They were dried in an oven at 65°C for 3 days and weighed using a balance.

The thiobarbituric acid method was used to determine the MDA concentration. The proline content was determined using the indenetrione-sulfosalicylic acid technique. The nitroblue tetrazolium (NBT) technique was used to measure SOD activity, and guaiacol was used to measure POD activity. These techniques are detailed in our earlier study (Dong et al., 2019). Excel (Microsoft 365MSO) was used to draw the bar chart. Relative chlorophyll content was determined using MultispeQV2.0.

### 2.3 Transcriptomics and metabolomics experimental methods

A total of 12 complementary DNA (cDNA) libraries were constructed (including three replicates of treatment 2 and 3). cDNA libraries were sequenced on an Illumina sequencing platform. The original data were filtered using fastp 0.19.3. HISAT v2.1.0 created an index and aligned clean reads to the reference genome. DESeq2 v1.22.1 was used to conduct a differential expression analysis between the two groups, and the Benjamini & Hochberg technique was applied to adjust the P value. For significant differential expression, the adjusted P value and |log2fold change| were used as criteria. |log2fold change| ≥ 1 and FDR <0.05 were the screening criteria for differentially expressed genes (DEGs). The hypergeometric test constituted the foundation for the enrichment analysis of genes with differential expression. The hypergeometric distribution test for Kyoto Encyclopedia of Genes and Genomes (KEGG) was carried out based on the route.

The preliminary preparation of metabolomics was carried out in accordance with established protocols at Metware Biotechnology Co., Ltd. (Wuhan, China). VIP ≥ 1 and |log2fold change| ≥ 1 were used to identify the significantly regulated metabolites between groups. Using the R package MetaboAnalystR, variable important in projection (VIP) values were extracted from the Orthogonal Partial Least Squares Discriminant Analysis (OPLS-DA) data, and score and permutation plots were produced. The data were mean-centered and logarithmically transformed (log2) before OPLS-DA. The statistical function prcomp in R performed unsupervised principal component analysis (PCA) (www.r-project.org). The discovered metabolites were annotated by utilizing the KEGG database. R (basic package) version 3.5.1, was used for PCA. OPLS-DA was performed using R (MetaboAnalystR; version 1.0.1). Cytoscape was used to create correlation networks.

### 2.4 Real-time quantitative polymerase chain reaction detection method

Norminkoda Biotechnology Co., Ltd (Wuhan, China) performed the qPCR detection. Utilizing MonScript™ RTIII All-in-One Mix with dsDNase, reverse transcription of whole RNA into cDNA. The primers used in qPCR are presented in **Supplementary Table S1**. The PCR process was performed as follows: First, the initial heat activation of PCR was performed at 90°C for two minutes for one cycle. Secondly, denaturation at 95°C for 5 seconds, combined annealing/extension at 60°C for 30 seconds, 40 cycles. Finally, the melting curve was analyzed. To determine the relative expression of the genes, the internal reference gene *ACT11* was used, and the 2^-ΔΔCt^ method was applied.

## 3 Results

### 3.1 MC reduced plant height but promoted dry matter accumulation under drought stress

**Figure 1:**
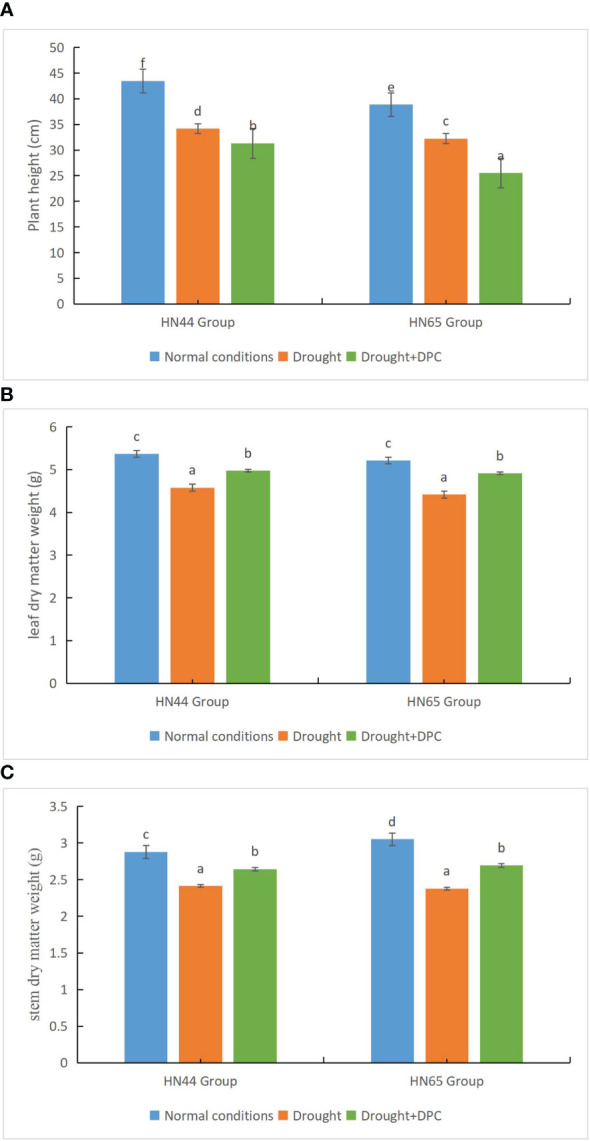
Evaluation of growth status among treatments. **(A)** Plant height. **(B)** Leaf dry weight. **(C)** Stem dry weight. Different letters in each variety represent 5 % significantdifference between treatments.

We first compared the growth of plants under the three treatment conditions, i.e., normal, drought stress, and drought + MC. Under normal conditions, the two plant varieties (HN44 and HN65) grew to a height of 43.5 cm and 38.9 cm, respectively. The heights of the two plant varieties drastically dropped under drought stress, to 34.2 cm and 32.2 cm, respectively. Under drought + MC treatment, the plant height further decreased to 31.3 cm and 25.5 cm, respectively, and the difference between the treatments was significant (**Figure 1A**). Additionally, the dry matter weight of leaves was significantly different among the treatments (**Figure 1B**). Under normal conditions, the leaf dry weight of the two varieties reached 5.37 g and 5.21 g, respectively. Under drought stress, the leaf dry weight of the two varieties decreased significantly, to 4.58 g and 4.42 g, respectively. Under drought + MC treatment, the leaf dry weight was higher than that under drought treatment, at 4.97 g and 4.91 g, respectively. The stem dry matter weight was also significantly different among the treatments (**Figure 1C**). The stem dry weights under normal conditions were 2.88 g and 3.05 g, while they drastically decreased to 2.41 g and 2.38 g, respectively, under drought stress. The stem dry weight was higher under the drought + MC treatment than under the drought treatment, at 2.64 g and 2.69 g, respectively, indicating that MC plays a specific role under drought stress conditions in promoting plant growth.

### 3.2 MC alleviates plant damage caused by drought stress

**Figure 2:**
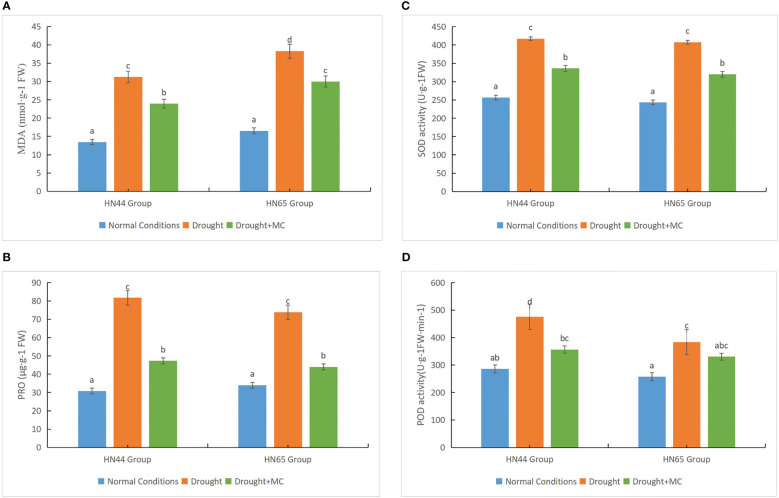
Drought resistance evaluation among different treatments. **(A)** malondialdehyde (MDA), **(B)** proline, **(C)** superoxide dismutase (SOD), and **(D)** peroxidase (POD). Different letters in each variety represent 5 % significantdifference between treatments.

We investigated the accumulation of MDA and proline and the activity of SOD and POD to elucidate the physiological impacts of MC on soybean under drought stress. Drought stress resulted in reduced accumulation of MDA in the two varieties compared to the normal water circumstances (**Figure 2A**); however, the MDA level in HN65 was higher, indicating more damage, compared with HN44. Further, the MDA concentration of the two varieties drastically decreased under the drought + MC treatment, and MC reduced membrane lipid peroxidation. Under drought stress, the proline contents of the two varieties greatly increased (**Figure 2B**), with HN44 having a greater proline content than did HN65. However, the proline content considerably decreased upon treatment with drought + MC, as MC drastically decreased proline accumulation. The SOD and POD activities of the two varieties drastically increased under drought stress, with the SOD activity being consistently higher in HN44 than in HN65 (**Figures 2C, D**). The POD activity of HN44 drastically decreased under the drought + MC treatment, while that of HN65 was reduced, although not significantly. In general, HN44 was more resistant to drought stress, and MC alleviated the damage caused by drought stress to some extent.

### 3.3 Transcriptomic data evaluation and differential gene identification

Twelve cDNA libraries (including three biological replicates for each treatment) were built and sequenced to know the response of the two soybean cultivars to drought stress. Each cDNA library produced between 58 million and 71 million raw reads, and between 56 million and 66 million clean reads, yielding 108.75 G clean reads (**Supplementary Table S2**). Each sample’s clean base was distributed between 8.45 and 9.99 G. Q30 bases accounted for more than 93.2% of the total base content, whereas GC bases accounted for between 44.53% and 45.67%. The comparison rate of each sample was above 96.2% when the clean reads were compared to the reference genome, demonstrating that the sequencing quality was good and the subsequent analysis could be performed.

Under drought stress, MC exhibited various impacts on the two soybean varieties. The HN44 variety had 2476 DEGs, including 674 downregulated and 1802 upregulated genes. The HN65 variety had 2752 DEGs, comprising 1430 downregulated and 1322 upregulated genes. The two varieties co-expressed 756 DEGs in total.

### 3.4 Functional analysis of the differential genes

To understand the functional profile of the differential genes, gene ontology annotation of the differential genes was performed (**Figure S1**). DEGs in HN44 were particularly abundant in several components involved in photosynthesis, including the chlorophyll biosynthesis process, chlorophyll metabolism process, light capture, photosynthesis, photosystem 1, pigment metabolism process, and response to high light intensity. In addition, other pathways, such as the synthesis and metabolism of vitamin E, were involved. DEGs in HN65 were mainly enriched in glucose metabolism, cell metal ion balance, cuticle development, fatty acid biosynthesis, fatty acid metabolism, lipid metabolism, photosynthesis, light reaction, photosynthetic electron transport chain, extracellular space photosystem, plant cell wall, iron ion binding, and other items. The application of MC affected the photosynthesis-related processes in the two varieties.

KEGG significant enrichment analysis was performed to further understand the metabolic pathways involved in DEGs (**Figure S2**). Photosynthesis, Photosynthesis-antenna proteins, Porphyrin and chlorophyll metabolism, Biosynthesis of secondary metabolites, and Plant-pathogen interaction were the top five most significantly enriched in HN44. In addition, plant-pathogen interaction, MAPK signaling pathway-plant, plant hormone signal transduction, glycosphingolipid biosynthesis-ganglio series, and biosynthesis of secondary metabolites were the top five most significantly enriched in HN65.

### 3.5 Effects of MC on the photosynthetic system under drought stress

**Figure 3:**
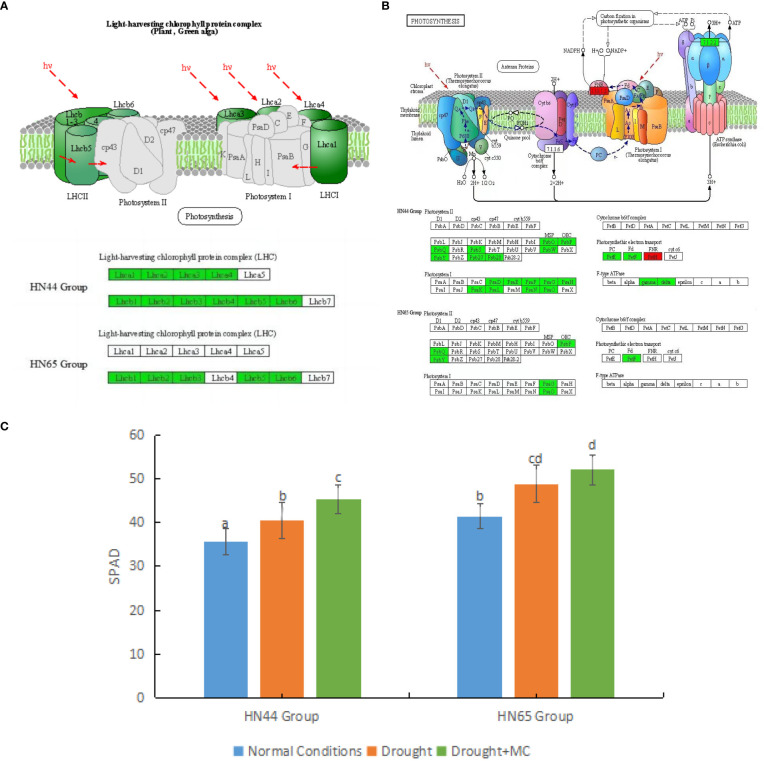
Effects of mepiquat chloride (MC) on the photosynthetic system under drought stress. **(A)** light capture system, **(B)** photosynthesis, and **(C)** relative chlorophyll content. Different letters in each variety represent 5 % significantdifference between treatments.

First, the light capture process in both varieties was affected (**Figure 3A**). In HN44, the genes encoding light-harvesting complex I proteins Lhca1, Lhca2, Lhca3, and Lhca4 were downregulated, and the genes encoding light-harvesting complex II proteins Lhcb1, Lhcb2, Lhcb3, Lhcb4, Lhcb5, and Lhcb6 were downregulated. In HN65, the genes encoding light-harvesting complex II proteins Lhcb1, Lhcb2, Lhcb3, Lhcb5, and Lhcb6 were downregulated. The downregulation of these proteins represents a decrease in the light capture efficiency of the two varieties.

MC pretreatment further inhibited soybean photosynthesis under drought stress (**Figure 3B**). Owing to the reduction of light capture efficiency, other processes were also affected. In HN44, MC had a stronger inhibitory effect on photosynthesis. In the photosynthetic system II, the genes encoding PsbO, PsbP, PsbQ, PsbS, PsbW, PsbY, Psb27, and Psb28 were downregulated. In the photosynthetic system I, the genes encoding photosynthetic subunits PsaD, PsaE, PsaF, PsaG, PsaH, PsaK, PsaL, PsaN, and PsaO were downregulated. In addition, the genes encoding plastocyanin and ferredoxin were downregulated in photosynthetic electron transport. In contrast, *LOC100777573* (Log2FC = 2.08), which only encodes NADP + reductase, was upregulated, while genes encoding ATPF1G and ATPF1D were downregulated. In HN65, the inhibitory effect of MC on photosynthesis was weaker than that in HN44. In photosystem I, only the genes encoding PsaG and PsaO were downregulated, whereas in photosystem II, all the genes encoding PsbP, PsabQ, and PsbY were downregulated, and all the genes encoding ferredoxin in photosynthetic electron transport were downregulated. The expression of PsbP, PsabQ family proteins, PsaG, and PsaO family proteins may be closely related to MC. We also observed an increase in relative chlorophyll content (**Figure 3C**). When the two soybean varieties were under drought stress, the relative chlorophyll content increased significantly. After MC application, the relative chlorophyll content increased significantly in HN44. However, the SPAD value of HN44 was generally lower than that of HN65.

### 3.6 Effects of MC on GA metabolism under drought stress

**Figure 4:**
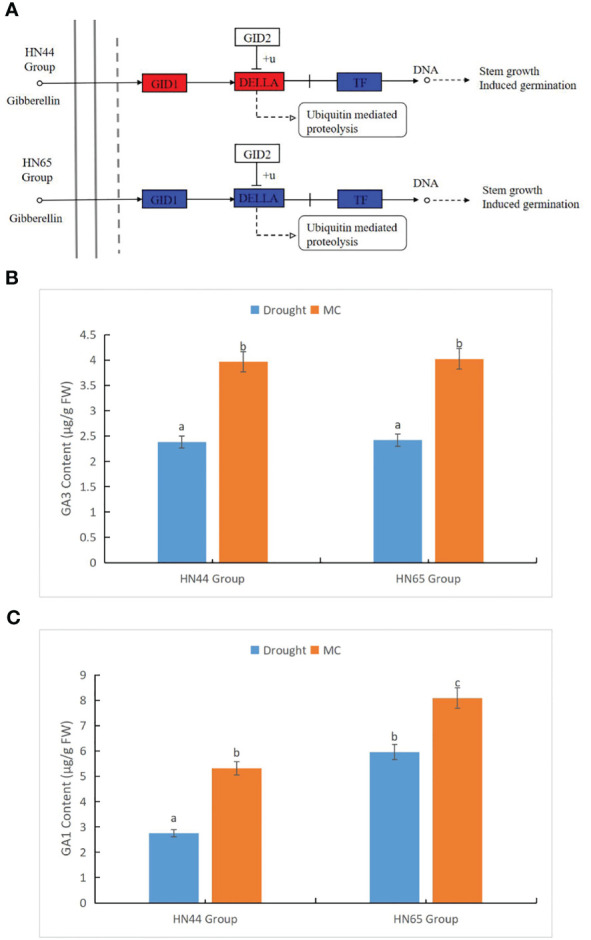
Effects of mepiquat chloride (MC) on gibberellin metabolism under drought stress. **(A)** gibberellin (GA) metabolic pathway, **(B)** GA3 content, and **(C)** GA1 content. Different letters in each variety represent 5 % significantdifference between treatments.

We further studied the effect of MC on GA metabolism under drought stress (**Figure 4A**). In the HN44 Group, nine genes were involved in this pathway. Three GID1 (GID1) -related genes, *LOC100806530* (Log2FC = 2.91, the same below), *LOC100779813* (2.18), and *LOC100788435* (1.65), were upregulated. Further, *LOC100796434* (1.05), encoding DELLA protein, was upregulated and affected by photosynthesis. On the contrary, the five genes encoding phytochrome-interacting factor 4 (PIF4) were only upregulated by *LOC100807098* (3.44), and the remaining four genes *LOC100784531* (-1.20), *LOC100798855* (-1.61), *LOC100814124* (-1.23), and *LOC100819517* (-1.74) were downregulated. In HN65, 16 genes were involved in this pathway; *LOC100812133* (1.43), *LOC106797394* (1.39), *LOC100779813* (1.54), and *LOC100788435* (1.92) encoding GID1 were upregulated, *LOC100795069* (-2.52) was downregulated, and *LOC100775665* (1.148) and *LOC100788103* (1.20) encoding DELLA protein were upregulated. In addition, only one of the nine genes encoding PIF4, *LOC100811345* (1.37), was upregulated. At the physiological level, we measured the content changes of some hormones in the GA family. GA 9, GA 15, GA 7, GA 20, GA 53, GA 4, and GA 24 were not detected, and GA 1, GA 19, and GA 3 content changed after MC application. The content of GAs 3 and 1 in the two varieties increased significantly after MC application under drought stress (**Figures 4B, C**), whereas the content of GA 19 decreased significantly. Under drought stress, the content of GA19 in HN44 was 0.73 µg/g FW, and the content in HN65 was 0.34 µg/g FW, whereas GA 19 was not detected after MC application. Overall, GA metabolism was enhanced at the transcriptional level, and at the physiological level, GAs 1 and 3 were also increased, thus maintaining stem growth under drought stress.

### 3.7 qRT-PCR validation

**Figure 5:**
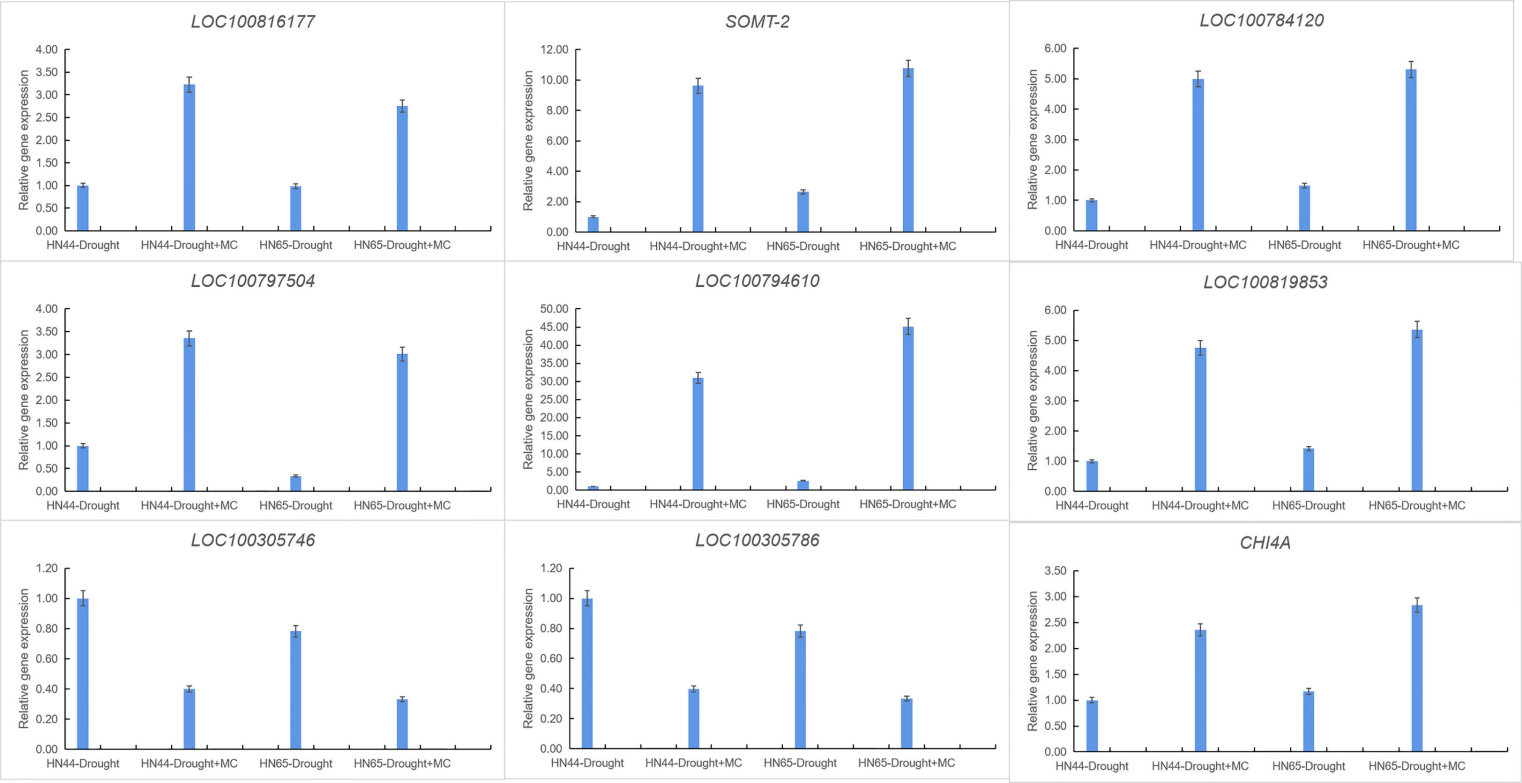
Gene expression was analyzed using quantitative real-time PCR compared to Drought as a control. The *ACT11* gene was used as an internal reference gene for expression normalization. The error bars indicate the SD of three qRT-PCR biological replicates.

Nine crucial genes were selected for qRT-PCR verification to confirm the transcriptome data’s validity (**Figure 5**). *LOC100816177* was involved in 2-oxocarboxylic acid metabolism, *SOMT-2*, *LOC100784120*, *LOC100797504*, *LOC100794610*, and *LOC100819853* were involved in isoflavone biosynthesis, *LOC100305746* and *LOC100305786* were involved in photosynthesis, and *CHI4A* was involved in flavonoid biosynthesis. The qPCR results of nine detected genes were consistent with the RNA-Seq expression trend. These results indicate the high reliability of RNA-Seq data.

### 3.8 PCA and OPLS-DA

**Figure 6:**
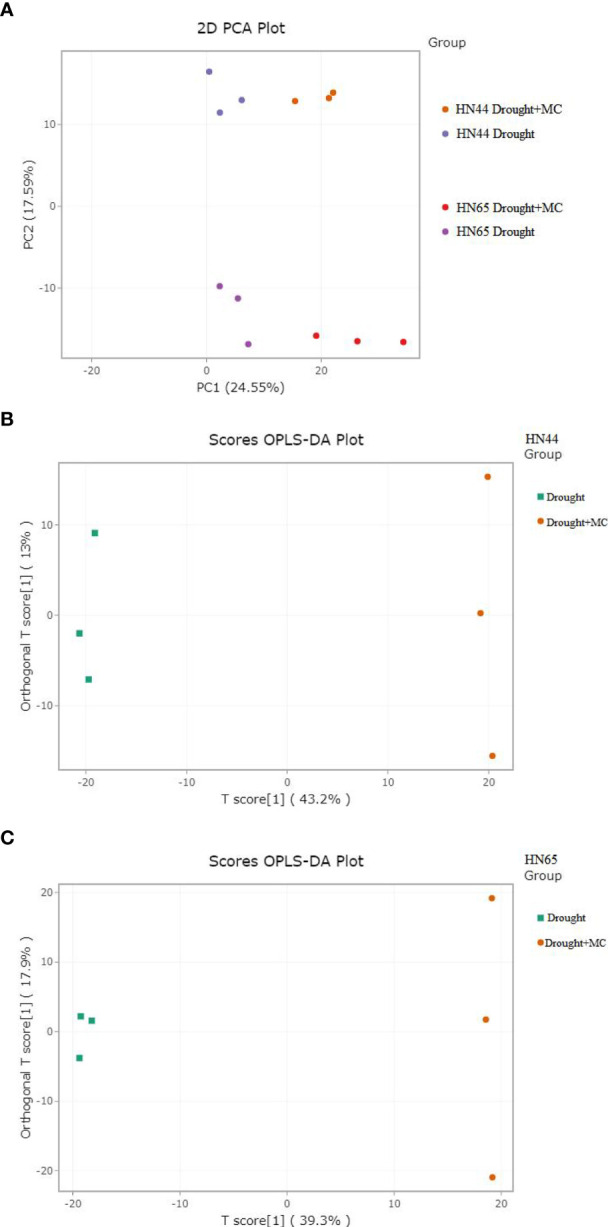
PCA and OPLS-DA score chart. **(A)** principal component analysis (PCA), **(B, C)** OPLS-DA.

PCA analysis was conducted on the samples. There was an evident distinction between the groups affected by drought and those affected by drought + MC (**Figure 6A**), indicating that MC application resulted in significant metabolic changes between the two varieties. A partial least squares regression was used to create a relationship model between metabolite expression and sample categorization. The outcomes demonstrated that R2X, R2Y, and Q2 were higher than 0.562, 0.846, and 0.936, respectively, across the four sets of samples, demonstrating the sensitivity of metabolites to drought stress (**Figure 6B, C**). The model was deemed to be accurate, because R2Y’ and Q2’ were both less than that of the original model. Differential metabolites were then examined using VIP value analysis.

### 3.9 Identification and classification of differential metabolites in the two varieties

**Figure 7:**
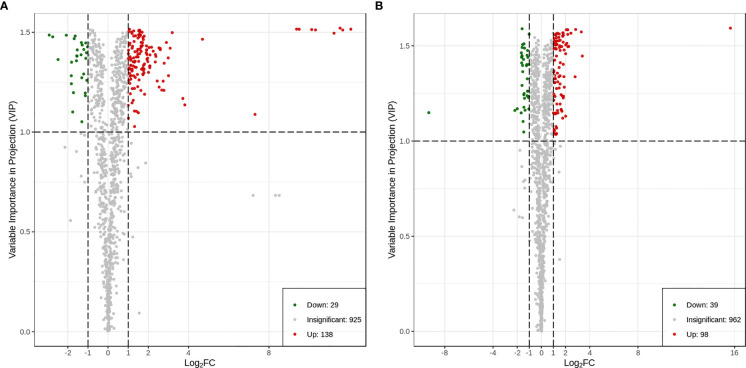
Volcano plot of differential metabolites. **(A)** HN44; **(B)** HN65.

A total of 167 differential metabolites (138 upregulated and 29 downregulated) were produced in HN44 (**Supplemental Table S3**), and 137 differential metabolites (98 upregulated and 39 downregulated) were produced in HN65 (**Supplemental Table S4**). **Figure 7** shows the volcano plot of paired comparison of differential metabolites.

### 3.10 Analysis of the global changes in the metabolic pathways

**Figure 8:**
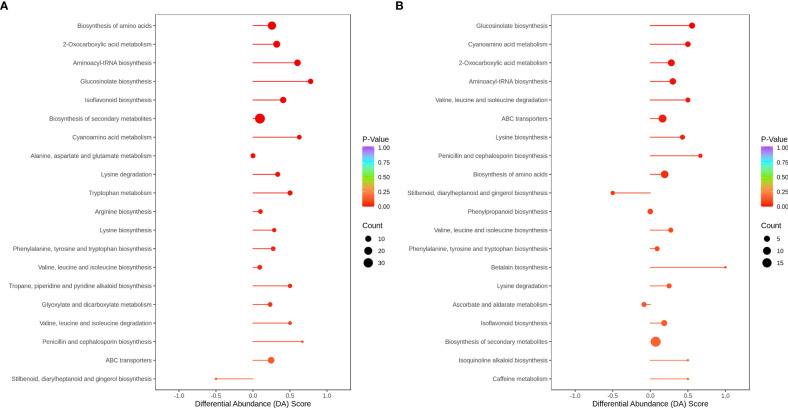
Differential abundance score plot for metabolites. **(A)** HN44; **(B)** HN65.

In HN44, only stilbenoid, diarylheptanoid, and gingerol biosynthesis were downregulated in the top 20 metabolic enrichment pathways, and the expression patterns of the remaining 19 pathways tended to be upregulated. Based on P values, differential metabolites were mainly enriched in the biosynthesis of amino acids, 2-oxocarboxylic acid metabolism, aminoacyl-tRNA biosynthesis, glucosinolate biosynthesis, and isoflavonoid biosynthesis. In HN65, only stilbenoid, diarylheptanoid and gingerol biosynthesis, and ascorbate and aldarate metabolism tended to be downregulated in the top 20 metabolic enrichment pathways, and the expression patterns of the remaining 18 pathways tended to be upregulated. Differential metabolites were mainly enriched in glucosinolate biosynthesis, cyanoamino acid metabolism, 2-oxocarboxylic acid metabolism, aminoacyl-tRNA biosynthesis, and valine, leucine, and isoleucine degradation. MC improved flavonoid and amino acid metabolism under drought stress in soybean (**Figure 8**).

### 3.11 MC-induced accumulation of flavonoids and amino acids under drought stress

MC made two cultivars accumulate a large amount of flavonoids and amino acids at metabolic level under drought stress. A total of 55 flavonoids (6 downregulated) were accumulated in HN44, 48 (8 downregulated) in HN65, and 24 flavonoids were detected between the two varieties. Except for the opposite expression of isorhamnetin-7-O-glucoside between the two varieties (upregulated in HN44 and downregulated in HN65), the other 23 flavonoids were upregulated. Among the accumulated substances, the Log2FC value of HN44 was also predominantly greater than that of HN65, and the Log2FC values of substances, such as prunetin-4’-O-glucoside and izalpinin, reached 10.12 and 9.5, respectively. In addition, the two varieties accumulated some unique flavonoids to resist drought stress, such as 7-hydroxy-4’-methoxyisoflavone, formononetin, and garbanzol in HN44, and chrysin, tectochrysin, and 3’-methoxydaidze in HN65. However, HN44 had more upregulated flavonoid metabolites.

Regarding amino acid accumulation, 31 (all upregulated) and 22 (two downregulated) amino acids and their derivatives were accumulated in HN44 and HN65, respectively; 18 substances and accumulation of certain common drought-resistant marker metabolites, such as L-proline and L-phenylalanine, were detected between the two varieties. Among the 18 accumulated amino acids, the Log2FC value of HN44 was greater than that of HN65. In addition, the two varieties accumulated certain amino acids for drought-stress resistance, such as L-lysine, L-histidine, and L-homomethionine in HN44, and N-acetyl-L-glycine, cyclo (Ala-Gly), and glycyl-tryptophan in HN65. However, HN44 had more upregulated amino acid metabolites.

### 3.12 Correlation analysis of genes and metabolites

**Figure 9:**
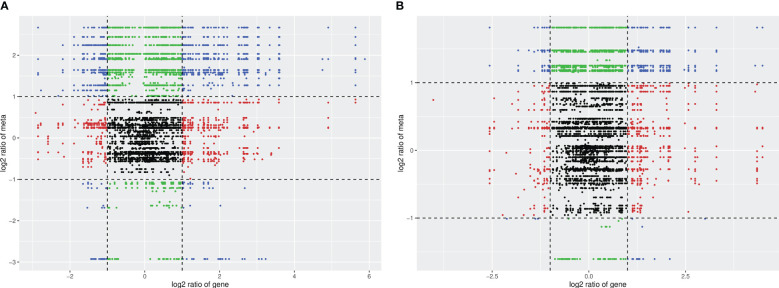
Correlation analysis nine-quadrant plot. **(A)** HN44; **(B)** HN65.

**Figure 9** indicates that the differences in the genes and metabolites of the two varieties are primarily distributed in the second, fourth, sixth, and eighth quadrants. The expression abundance of metabolites was higher than that of genes in the second and fourth quadrants. The metabolites were upregulated, whereas the genes were either unchanged or downregulated. In the sixth and eighth quadrants, the expression abundance of metabolites was lower than that of genes; the genes were upregulated, whereas the metabolites were unchanged or downregulated. Under the drought stress + MC treatment, the transcription and metabolism levels in HN44 increased, the transcription level in HN65 was slightly inhibited, and the metabolism level increased.

### 3.13 Enrichment pathways of differential genes and metabolites

**Figure 10:**
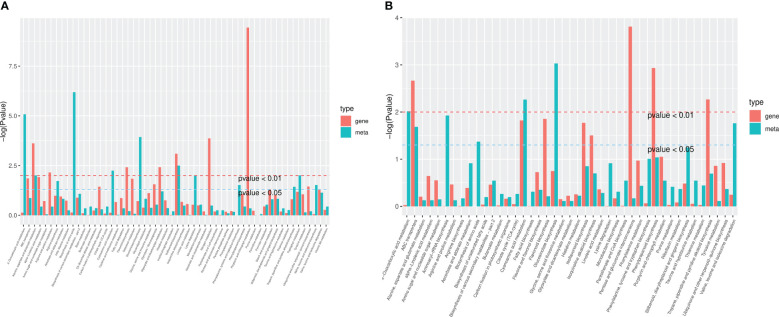
Kyoto encyclopedia of genes and genomes (KEGG) enrichment plot of differential genes and metabolites. **(A)** HN44; **(B)** HN65.

The differential genes and metabolites of the same group were mapped to the KEGG pathway map simultaneously to better understand the relationship between genes and metabolites (**Figure 10**). In HN44, differential genes and metabolites were involved in 56 pathways, mainly enriched in the biosynthesis of amino acids, 2-oxocarboxylic acid metabolism, glucosinolate biosynthesis, isoflavonoid biosynthesis, cyanoamino acid metabolism, and other pathways. In HN65, differential genes and metabolites were involved in 43 pathways, mainly enriched in glucosinolate biosynthesis, cyanoamino acid metabolism, 2-oxocarboxylic acid metabolism, aminoacyl-tRNA biosynthesis, and valine, leucine, and isoleucine degradation.

### 3.14 Amino acid metabolic system cascaded by 2-oxyacids

**Figure 11:**
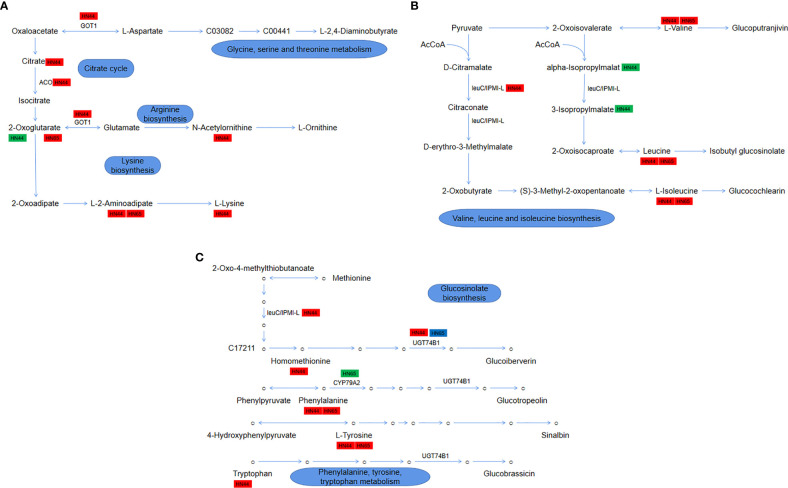
**(A)** Module 1: glycine, serine, and threonine metabolism, citrate cycle, arginine biosynthesis, and lysine biosynthesis; **(B)** Module 2: valine, leucine, and isoleucine biosynthesis; **(C)** Module 3: glucosinolate biosynthesis and phenylalanine, tyrosine, and tryptophan metabolism.

The drought stress + MC treatment activated the 2-oxocarboxylic acid metabolism pathway, which involves the metabolism/synthesis of various amino acids and plays an essential role in the drought resistance of soybean. In HN44, 5 genes and 14 metabolites were involved in this pathway; in HN65, 3 genes and 7 metabolites were involved. As shown in **Figure 11A**, it mainly involves four parts: glycine, serine, and threonine metabolism, citrate cycle, arginine biosynthesis, and lysine biosynthesis. In HN44, MC application led to the accumulation of citrate (1.33), and *LOC100778506* (3.05) encoding aconitate hydratase (ACO) was upregulated to promote the conversion of citric acid to isocitric acid. α-ketoglutarate (-2.49) was downregulated owing to the upregulation of *AAT* (1.62) encoding aspartate aminotransferase (GOT1), which accelerated the metabolism of α-ketoglutarate, and its downstream precursor for ornithine synthesis, N-acetylornithine (1.75), was also accumulated. In lysine synthesis, the content of L-alpha-aminoadipate (2.24) increased, which is mainly involved in the AAA pathway (2-ketoglutarate = > 2-aminoadipic acid = > lysine), leading to the accumulation of lysine (1.53). In HN65, only two metabolites, α-ketoglutarate (1.55) and L-alpha-aminodipate (1.48), were involved; as shown in **Figure 11B**, valine, leucine, and isoleucine biosynthesis were mainly involved. *LOC100811656* (2.95), which encodes the IPMI-L in HN44, was upregulated to promote the biosynthesis of leucine and isoleucine, which also resulted in the accumulation of both leucine (1.64) and isoleucine (1.59) and the increase of valine (1.44) content. In addition, two metabolic intermediates, 2-isopropylmalic acid (-1.54) and 3-isopropylmalate (-1.56), were downregulated. In HN65, only three metabolites were accumulated, among which, leucine (1.19), isoleucine (1.19), and valine (1.22) were upregulated, and no gene changes were observed. Although these three amino acids were accumulated between the two varieties, the fold change of HN44 was greater. As shown in **Figure 11C**, glucosinolate biosynthesis and phenylalanine, tyrosine, and tryptophan metabolism are mainly involved. Four amino acids, homomethionine (1.09), tryptophan (1.79), tyrosine (1.27), and phenylalanine (1.57), were accumulated in HN44 after MC application. In addition, two genes encoding N-hydroxythioamide S-beta-glucosyltransferase (UGT74B1), *LOC100783133* (1.28) and *LOC100816177* (1.48), were upregulated to promote homomethionine and tryptophan metabolism. In HN65, two amino acids, phenylalanine (1.20) and tyrosine (1.19), were upregulated, and *LOC100786887* (-2.15) encoding phenylalanine N-monooxygenase (CYP79A2) was downregulated. Between the two genes encoding UGT74B1, *LOC100816177* (1.40) was upregulated and *LOC100783761* (-1.04) was downregulated. Generally, HN44 exhibited more types of metabolites and genes, and the upregulation factor was generally greater than that of HN65. These genes/metabolites play an important role in drought resistance.

### 3.15 Isoflavone-dominated antioxidant system

**Figure 12:**
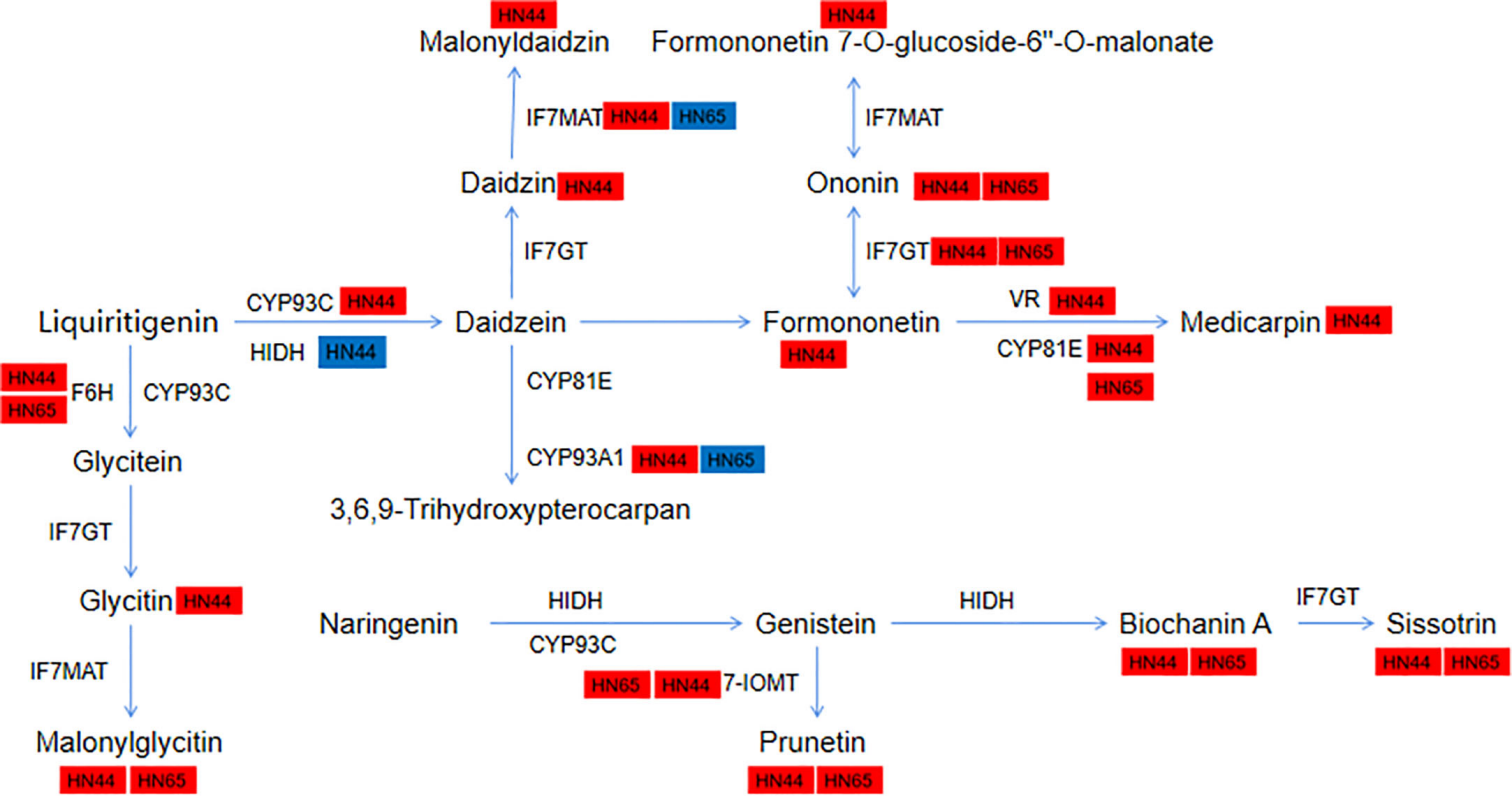
Changes of genes and metabolites in the isoflavone biosynthetic pathway.

Pre-application of MC led to the “burst” of isoflavone synthesis under drought stress. A total of 11 metabolites and 19 genes were involved in HN44, and 5 metabolites and 15 genes were involved in HN65. We further annotated the genes and metabolites encoding various enzymes in this pathway (**Figure 12**). In HN44, *IFS2* (3.26) and *novel-550* (3.51) encoding 2-hydroxyisoflavanone synthase (CYP93C) were upregulated, *HIDH* (1.63) was upregulated, and *LOC100817797* (-1.16) was downregulated. *LOC100819853* (1.60) and *SOMT-2* (2.89) encoding isoflavone-7-O-methyltransferase (7-IOMT) were upregulated, and two isoflavone 7-O-glucosyltransferase (IF7GT) genes *GMUGT4* (1.30) and *LOC100775378* (3.28) were upregulated. This increased the content of metabolites in the middle and lower reaches of this pathway, mainly including prunetin (1.42), biochanin A (1.32), and sissotrin (1.76). In addition, the flavonoid 6-hydroxylase (F6H) gene *LOC100797504* (1.51) was upregulated, and the encoding isoflavone 7-O-glucoside-6”-O-malonyltransferase (IF7MAT) four genes *IF7MaT* (1.10), *LOC100813644* (3.84), *LOC100814826* (1.90), and *LOC100815354* (1.14) were upregulated, and its downstream metabolites glycitein 7-O-glucoside (1.20) and malonylglycitin (4.69) were accumulated. In addition, formononetin 7-O-glucoside (2.53) and formononetin 7-O-glucoside-6” -O-malonate (2.16) were upregulated downstream of formononetin (3.00). In the daidzein = > medicarpin pathway, two vestitone reductase (VR) genes *LOC100194416* (2.08) and *LOC547660* (2.22) and three CYP81E genes *CYP81E11* (2.15), *LOC100784120* (1.68), and *LOC100794610* (5.00) were upregulated, eventually leading to the accumulation of medicarpin (7.31). Two metabolites downstream of daidzein, daidzein 7-O-glucoside (1.25), malonyldaidzin (1.81), and *CYP93A1* (4.12) encoding 3,9-dihydroxypterocarpan 6a-monooxygenase, were upregulated. In HN65, five upregulated metabolites ononin (1.03), malonylglycitin (1.16), prunetin (1.33), biochanin A (1.16), and sissotrin (1.82) were detected in HN44. Other genes, including *CYP71D10* (1.87), *LOC100797504* (2.96), and *LOC100816377* (1.77) encoding the enzyme F6H and two 7-IOMT genes *LOC100819853* (1.27) and *SOMT-2* (1.43) were also upregulated. *GMUGT4* (1.39) and *LOC112999750* (1.91) encoding IF7GT and *LOC100784120* (1.54), *LOC100790507* (7.10), and *LOC100794610* (3.45) encoding CYP81E were upregulated. Unlike in HN44, *IF7MaT* (1.09) and *LOC100814826* (1.33) encoding IF7MAT were upregulated, and *LOC100801403* (-1.07) was downregulated in HN65. *LOC100807166* (1.10) encoding CYP93A1 was upregulated and *CYP76O2* (-1.89) was downregulated in HN65. Overall, HN44 has a greater variety of upregulated metabolites and genes. Some core genes and metabolites of F6H, 7-IOMT, IF7GT, and CYP81E, such as prunetin and biochanin A, may be closely related to MC.

### 3.16 Correlation network of genes and metabolites

**Figure 13:**
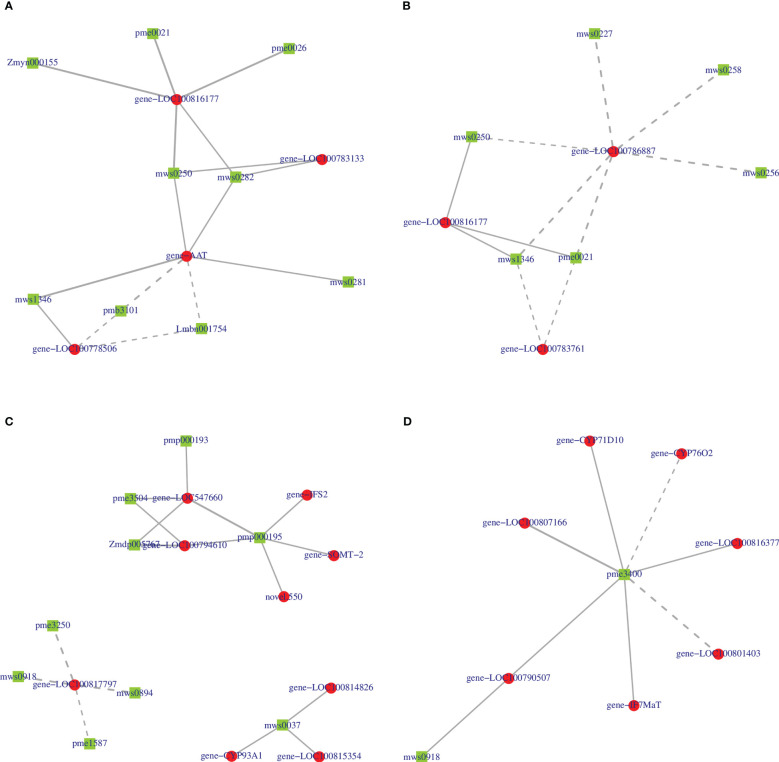
**(A)** 2-oxocarboxylic acid metabolism of HN44; **(B)** 2-oxocarboxylic acid metabolism of HN65; **(C)** isoflavonoid biosynthesis of HN44; **(D)** isoflavonoid biosynthesis of HN65.

We further constructed a correlation network of 2-oxocarboxylic acid metabolism (ko01210) and isoflavonoid biosynthesis (ko00943) for the two varieties to explain the correlation between metabolites and genes. In the ko01210 pathway, nine metabolites of HN44 regulated this pathway by targeting four genes *LOC100816177*, *LOC100783133*, *AAT*, and *LOC100778506* (**Figure 13A**), of which, 2-isopropylmalic acid and 3-isopropylmalic acid were negatively correlated with *AAT* and *LOC100778506*, and the remaining seven metabolites were positively correlated with the target gene. Six metabolites in HN65 targeted three genes, *LOC100786887*, *LOC100816177*, and *LOC100783761* (**Figure 13B**), of which, only tyrosine, DL-2-aminoadipic acid, and phenylalanine were positively correlated with *LOC100816177*, and all six metabolites were negatively correlated with *LOC100786887* and *LOC100783761*. In the ko00943 pathway, nine genes in HN44 formed three sub-networks with nine metabolites (**Figure 13C**). First, four metabolites, biochanin A, prunetin, daidzin, and glycitin, were negatively correlated with *LOC100817797*, which explained the downregulation of *LOC100817797* in HN44. Second, three genes were positively correlated with formononetin. Third, five genes were positively correlated with 6”-O-malonylglycitin. In addition, 6”-O-malonyldaidzin, nonin, and formononetin-7-O-(6”-malonyl) glucoside was positively correlated with *LOC547660* and *LOC100794610*. In HN65, seven genes and two metabolites formed a correlation network (**Figure 13D**). With sissotrin as the target, five genes were positively correlated, *CYP76O2* and *LOC100801403* were negatively correlated, and prunetin was positively correlated with *LOC100790507*. In summary, multiple genes target one metabolite, or multiple metabolites target one gene to regulate these pathways. Therefore, clarifying the correlation between differential genes and metabolites is essential for screening biomarkers. Based on the soybean variety, core genes such as *LOC100816177*, *AAT*, and *LOC100786887* and core metabolites such as prunetin, biochanin A, and sissotrin are closely related to MC.

### 3.17 Construction of comprehensive regulation network of MC regulation of soybean drought response

**Figure 14:**
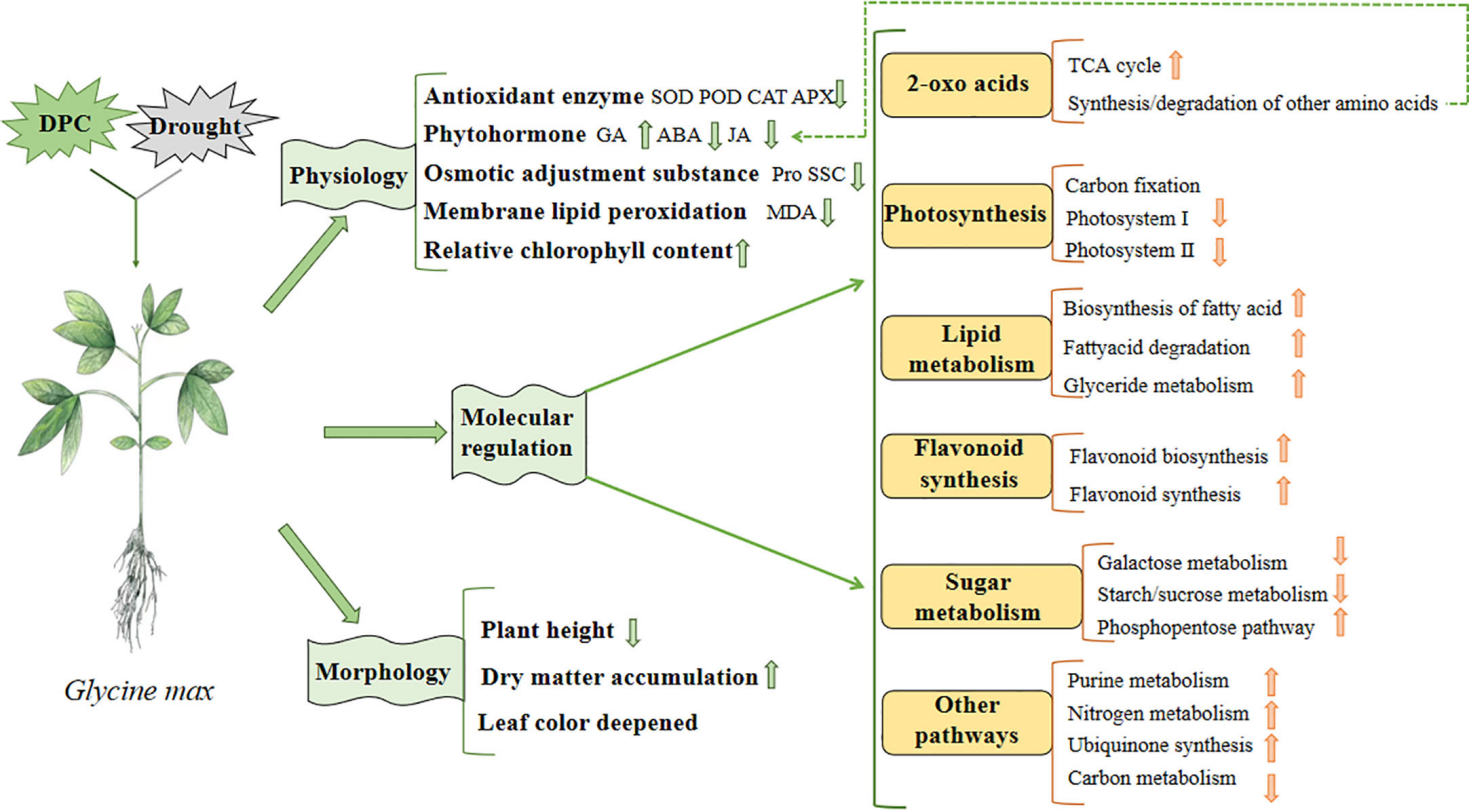
Model diagram of mepiquat chloride (MC) regulating soybean in response to drought.

Based on our previous studies, combined with physiological and omics data, we constructed a model to illustrate the effects of MC pretreatment on soybean under drought stress (**Figure 14**). Regarding growth, compared with the drought treatment, plant height decreased; however, in terms of physiology, dry matter accumulation increased, leaf color deepened, antioxidant enzyme activity decreased, ABA and jasmonic acid (JA) activity decreased, GA activity increased, proline content decreased, membrane lipid peroxidation weakened, and relative chlorophyll content increased. Furthermore, regarding molecular regulation, the TCA cycle, amino acid synthesis, flavonoid synthesis, fat metabolism, purine metabolism, nitrogen metabolism, ubiquinone synthesis, and pentose phosphate pathway were enhanced; however, photosynthesis and starch and sucrose metabolism were inhibited.

## 4 Discussion

MC is the most widely used growth regulator worldwide. It exhibits efficient internal absorption and conduction effects in plants and has a certain regulatory effect on the geometric shape of plants (Rosolem et al., 2013). With increasing research, cumulative functions of MC in plant production have been discovered. Han et al. (2009) reported that MC application increased chlorophyll content, root activity, and peroxidase activity of tall fescue seedlings. MC promoted seedling growth and enhanced seedling resistance. Du et al. (2014) reported that MC increased the POD activity of the roots of five sweet potato varieties under drought stress and reduced the MDA mass molar concentration of the roots of each sweet potato variety. In our study, the application of MC reduced plant height but increased dry matter accumulation under drought conditions (**Figure 1**) and reduced the contents of SOD, POD, MDA, and proline (compared with the drought treatment) (**Figure 2**). According to Lü et al. (2019), the level of drought stress and antioxidant enzyme activity are correlated to some extent. With increasing stress levels, antioxidant enzyme activity increases within a certain range. Regardless of the increase or decrease, we believe that the activity of antioxidant enzymes observed in this study is reasonable compared to that of other studies. Plant drought resistance is correlated with its capacity to maintain the equilibrium of active oxygen metabolism under drought stress. Following drought treatment, plants exhibited higher levels of ROS, and antioxidant enzyme activity surged instantaneously. After exogenous MC application, drought resistance was further improved. The drought resistance pathway (such as the accumulation of flavonoids) and the ROS scavenging ability were further increased. When the level of ROS decreases to reach a dynamic equilibrium, the activity of antioxidant enzymes gradually decreases. Similarly, the decrease in MDA level also represented the decrease in drought degree. In addition, antioxidant enzyme activity is affected by several factors, including stress time and stress degree.

Photosynthesis is a multi-step, tightly controlled process (Tanaka and Makino, 2009). It is especially crucial during the growth and development of the plant because it contributes the only carbon source vital for plant growth (Nowicka et al., 2018). Lack of water results in various plant reactions, restricting plant growth, development, and productivity. At various phenological stages, this morphological and physiological reaction is discernible (Mukarram et al., 2021a). Furthermore, a majority of environmental disturbances lead to increased ROS production. Under typical or non-stressful circumstances, a majority of plants maintain stable homeostasis. In contrast, several plants in various habitats have been observed to accumulate ROS owing to drought stress, and the increased ROS level under stress conditions is significant. However, ROS levels beyond plant quenching capacity can destroy nucleic acids, photosynthetic pigments, proteins, and membrane lipids, causing oxidative stress (Corpas et al., 2020; Mukarram et al., 2021b). Thus, photosynthesis is typically suppressed when plants are under stress. Previous studies have reported that MC inhibits photosynthesis, primarily by lowering the net photosynthetic rates of plants, although it also increases the amount of chlorophyll (chlorophyll a + b). The significant increase in chlorophyll caused by MC application did not lead to a higher photosynthetic rate (Tung et al., 2019), and there is no reasonable explanation for this situation. In our previous studies, we also noticed downregulation of light-harvesting proteins LHCA1, LHCA2, LHCB1, and LHCB2, as well as suppression of PsaO, PsaG, and PsaN in photosystem I, and PsbS in photosystem II (Wang et al., 2022c). Under the drought stress + MC treatment, the photosynthetic system was further inhibited, including stronger inhibition of light capture and inhibition of photosystems I and II, which was more severe in the drought-resistant variety HN44. Simultaneously, we noticed an increase in relative chlorophyll content (**Figure 3**). Based on the above experimental results, we speculated that this situation may be due to “a compensation effect” or “negative feedback regulation mechanism.” However, the increased chlorophyll content cannot compensate for decreased photosynthesis.

Drought can reduce annual agricultural yields more substantially than do all diseases combined. Plants modify their physiology, change the growth and structure of their roots, and close their aerial stomata to adjust to water gradients in the soil. These tissue-specific reactions change the transmission of cellular signals, which can cause early blooming or developmental delays and, frequently, poorer yield. Plant hormone signaling is key to controlling responses to drought or water deficiency, according to physiological and molecular studies of the model plant *Arabidopsis thaliana* (Gupta et al., 2020). Nir et al. (2014) revealed that decreasing GA levels or signaling increases plant resistance to environmental stressors. GA content decreases with an increase in drought degree. Therefore, the GA level can also reflect the degree of drought to a certain extent (Zhou et al., 2022). In previous studies, MC was used as a GA inhibitor in plant production (Wang et al., 2014), which reduced GA concentration and interfered with cell movement, resulting in cell wall relaxation (Yang et al., 1996). Therefore, MC can potentially impact plant drought resistance. In this study, notably, GA level under drought stress + MC application treatment was higher than that under drought treatment (GA1 and GA3), possibly because MC alleviated drought stress, thus increasing GA levels. However, this is contradictory to its inhibitory effect on GA biosynthesis; thus, the emergence of this phenomenon cannot be clearly explained. Incidentally, this study is the first to report the inhibitory effect of MC on GA19 under drought stress.

2-oxocarboxylic acids connect various amino acid metabolism/synthesis pathways, including the tricarboxylic acid cycle, and play an important role in plant drought resistance. Yang et al. (2022), who examined sugarcane response to drought, reported that 2-oxocarboxylic acid metabolism was the most important enrichment pathway. In this study, 2-oxocarboxylic acid metabolism was found to play an essential role in both soybean varieties. MC application under drought further activated this pathway in the two varieties (**Figure 11**). Some amino acids such as valine, leucine, isoleucine, phenylalanine, and tyrosine, and the enzymes related to amino acid synthesis/metabolism were upregulated. Flavonoids, which significantly accumulate under various abiotic stress conditions, are closely related to phenylalanine or tyrosine (Heinemann and Hildebrandt, 2021). Here, HN44 has more upregulated metabolites and genes in this pathway, such as citric acid and lysine. Citric acid mediates the TCA cycle, and lysine has also been shown to play an important role in plant stress (Wang et al., 2022e). You et al. (2019) reported that drought-resistant sesame showed high arginine, ABA, proline, and lysine levels under drought stress, highlighting the important role of amino acid metabolism in sesame drought tolerance. The difference in drought resistance between the two varieties can be attributed to these amino acids, which imparted higher drought tolerance in HN44.

Flavonoids are secondary metabolites found in plants; they are essential components of numerous biological processes and for the reactions of plants to their environment (Shen et al., 2022). Pre-application of MC caused a large accumulation of flavonoids under drought stress, of which, isoflavones accounted for the largest proportion (27/55 in HN44 and 15/48 in HN65). Isoflavones are a unique and important subclass of flavonoid compounds, mainly found in legumes, especially soybeans (Brodowska, 2017). They exhibit strong antioxidant properties and certain scavenging properties for free radicals. Additionally, they serve as antibacterial phytoalexins and regulate rhizosphere microbial communities to influence plant growth and crop yield formation (Weston and Mathesius, 2013; Sugiyama et al., 2017; Wang et al., 2018). These flavonoids scavenge a part of the ROS produced under drought stress, thus reducing the accumulation of ROS in plants; this may explain the lower antioxidant enzyme activity in the drought + MC treatment than that in the drought treatment. In HN44, these flavonoids had higher FC value and scavenging ability for ROS. The pre-application of MC further allocated more flavonoids in the two varieties, and accumulation of substances further improved the level of drought resistance.

Finally, we combined physiology, morphology, transcriptomics and metabolomics data to build a model to demonstrate the regulatory effect of MC on soybean growth and stress resistance under drought stress (**Figure 14**). Compared with the drought treatment, MC reduced the antioxidant enzyme activity and membrane lipid peroxidation, promoted soybean growth, and affected several pathways including 2-oxoacid metabolism, photosynthesis, fat metabolism, flavonoid synthesis, sugar metabolism, and nitrogen metabolism, under drought stress. In the future, we intend to reveal the regulatory effect of MC on soybean under drought stress by proteomics technology, to further enrich the regulatory mechanism of MC, to supplement the places not shown in the model, and finally to complete the construction of comprehensive regulatory network.

## 5 Conclusion

By combining physiological and omics techniques, we reported the mechanism of MC regulation of soybean response to drought. MC greatly alleviated plant damage caused by dehydration and decreased membrane lipid peroxidation. In addition, it scavenged excessive ROS by promoting the accumulation of amino acids and flavonoids, thereby improving the drought resistance of plants. Our study provides a theoretical basis for the use of MC in soybean and reveals the molecular mechanism of MC regulation of soybean drought response.

## Data availability statement

The NCBI database contains the datasets used in this study. The repository names and accession numbers are https://www.ncbi.nlm.nih.gov/, PRJNA854192 and PRJNA823397.

## Author contributions

XW wrote the manuscript, XZ and ZQ helped conduct physiological experiments, CY and CM supported the experiments, and JL and SD helped review the manuscript. All authors contributed to the article and approved the submitted version.
