# Supplementary figures and images for: Regulation of soybean drought response by mepiquat chloride pretreatment
Source: Front Plant Sci. 2023 May 8;14:1149114. doi: 10.3389/fpls.2023.1149114 (PMC10207941; doi:10.3389/fpls.2023.1149114)

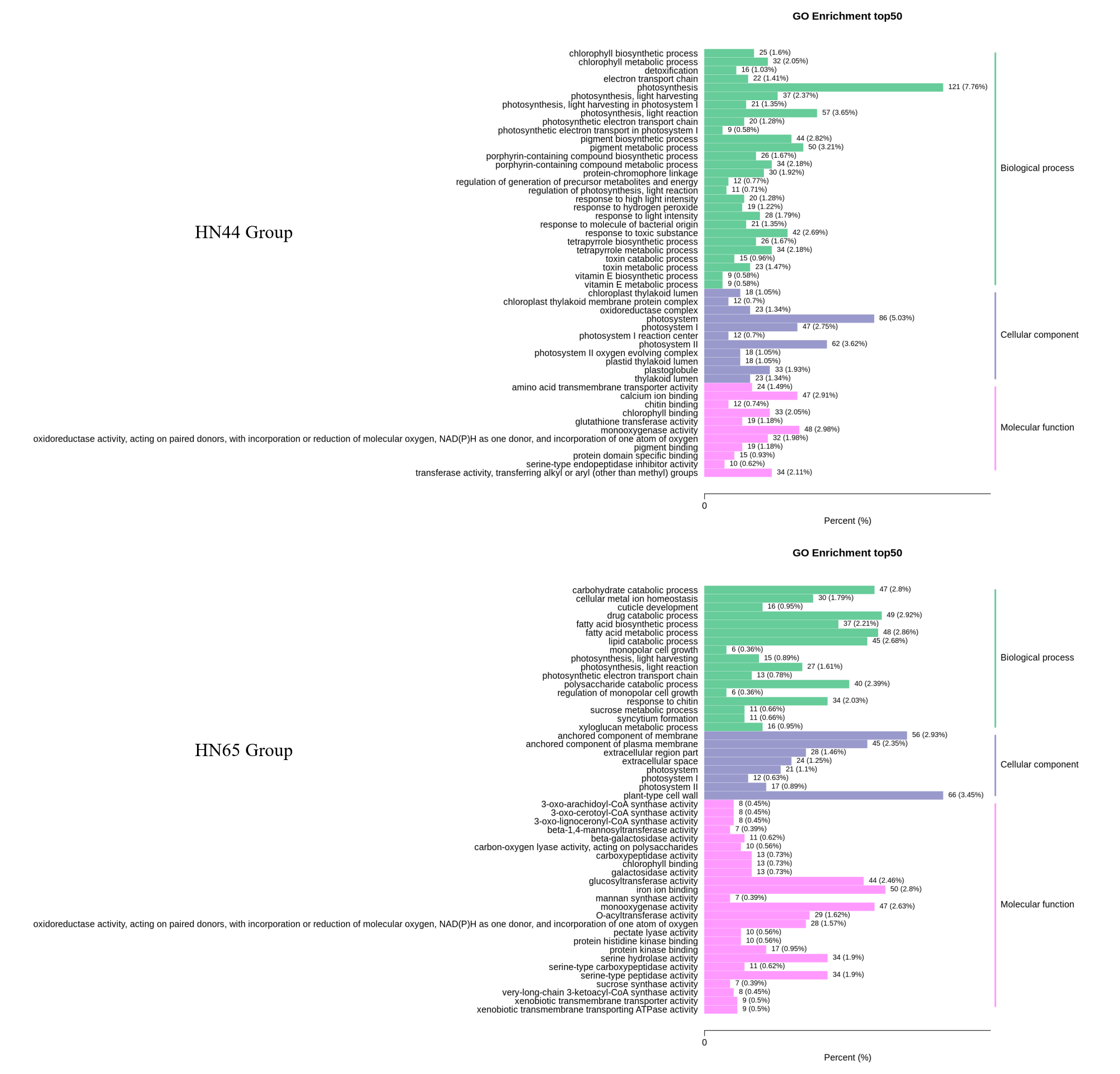

Supplement: Supplementary file 5 [file Image_1.png]

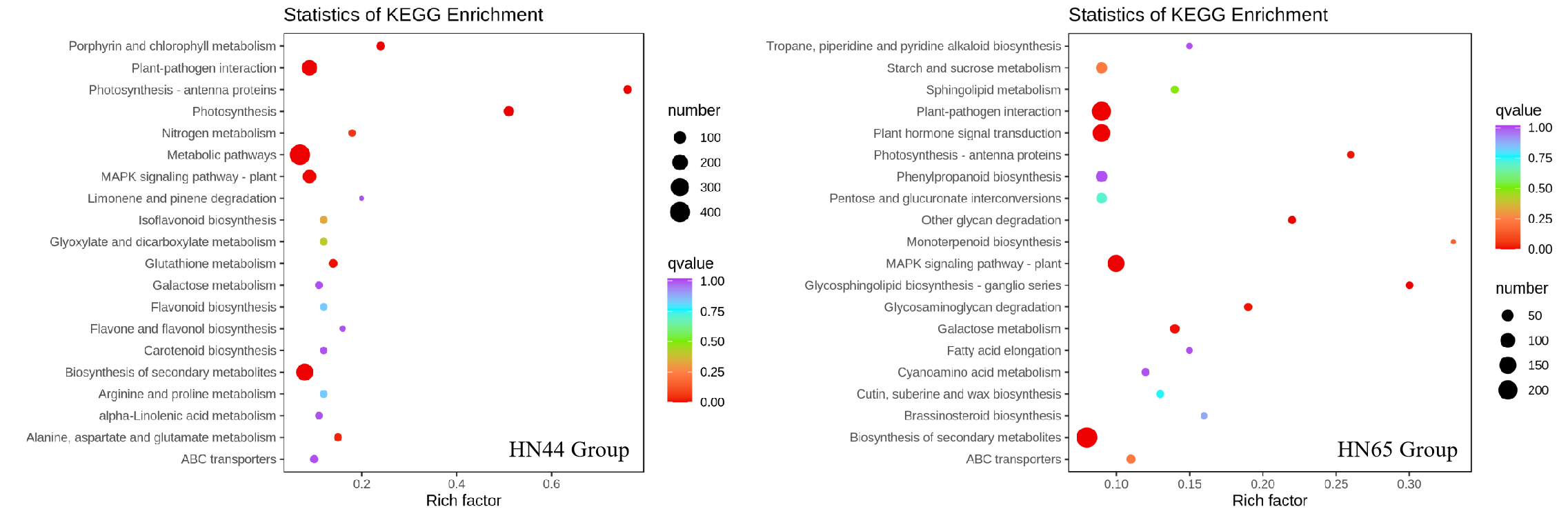

Supplement: Supplementary file 6 [file Image_2.png]
